# Effect of Aluminium Powder on Kaolin-Based Geopolymer Characteristic and Removal of Cu^2+^

**DOI:** 10.3390/ma14040814

**Published:** 2021-02-08

**Authors:** Nurliyana Ariffin, Mohd Mustafa Al Bakri Abdullah, Przemysław Postawa, Shayfull Zamree Abd Rahim, Mohd Remy Rozainy Mohd Arif Zainol, Ramadhansyah Putra Jaya, Agata Śliwa, Mohd Firdaus Omar, Jerzy J. Wysłocki, Katarzyna Błoch, Marcin Nabiałek

**Affiliations:** 1 Centre of Excellence Geopolymer and Green Technology (CEGeoGTech), Universiti Malaysia Perlis (UniMAP), Perlis 01000, Malaysia; nurliyana.arifin@gmail.com (N.A.); shayfull@unimap.edu.my (S.Z.A.R.); 2 Faculty of Chemical Engineering Technology, Universiti Malaysia Perlis (UniMAP), Perlis 01000, Malaysia; firdausomar@unimap.edu.my; 3 Department of Physics, Faculty of Production Engineering and Materials Technology, Częstochowa University of Technology, 42-201 Częstochowa, Poland; wyslocki.jerzy@wip.pcz.pl (J.J.W.); katarzyna.bloch@wip.pcz.pl (K.B.); nmarcell@wp.pl (M.N.); 4 Faculty of Mechanical Engineering Technology, Universiti Malaysia Perlis (UniMAP), Perlis 01000, Malaysia; 5 School of Civil Engineering, Engineering Campus, Universiti Sains Malaysia (USM), Pulau Pinang 14300, Malaysia; ceremy@usm.my; 6 Department of Civil Engineering, College of Engineering, Universiti Malaysia Pahang (UMP), Pahang 26300, Malaysia; ramadhansyah@ump.edu.my; 7 Division of Materials Processing Technology and Computer Techniques in Materials Science, Silesian University of Technology, 44-100 Gliwice, Poland; agata.sliwa@polsl.pl

**Keywords:** geopolymer, adsorbent, copper, foaming agent

## Abstract

This current work focuses on the synthesis of geopolymer-based adsorbent which uses kaolin as a source material, mixed with alkali solution consisting of 10 M NaOH and Na_2_SiO_3_ as well as aluminium powder as a foaming agent. The experimental range for the aluminium powder was between 0.6, 0.8, 1.0 and 1.2wt%. The structure, properties and characterization of the geopolymer were examined using X-Ray Diffraction (XRD), Infrared Spectroscopy (FTIR) and Scanning Electron Microscopy (SEM). Adsorption capacity and porosity were analysed based on various percentages of aluminium powder added. The results indicate that the use of aluminium powder exhibited a better pore size distribution and higher porosity, suggesting a better heavy metal removal. The maximum adsorption capacity of Cu^2+^ approached approximately 98%. The findings indicate that 0.8% aluminium powder was the optimal aluminium powder content for geopolymer adsorbent. The removal efficiency was affected by pH, adsorbent dosage and contact time. The optimum removal capacity of Cu^2+^ was obtained at pH 6 with 1.5 g geopolymer adsorbent and 4 h contact time. Therefore, it can be concluded that the increase in porosity increases the adsorption of Cu^2+^.

## 1. Introduction

Copper is one of the heavy metals that is hazardous to human health and the environment [1,2]. The descriptor heavy metal applies to any metal or metalloid material with a density between 3.5 and 7 g/cm^3^ [3,4,5]. This type of metal is commonly found in the Earth’s crust and is non-biodegradable. Due to the characteristics of high toxicity, biodegradation and bioaccumulation, heavy metals affect the aquatic organisms even at low concentrations [1,6]. In addition, heavy metals can also enter into human bodies through the food chain, resulting in serious threats to aquatic organisms and human health [6,7,8]. The main sources of copper commonly come from mining works, automobiles, and metal industries. Even if the amount of heavy metals such as Cu^2+^ is very minimal, the water is very poisonous to the body, especially if it reaches the maximum discharge level [9,10]. Therefore, many studies had been conducted on the decontamination of Cu^2+^ from wastewater [11,12,13,14]. Zeolites are microporous, aluminosilicate minerals commonly used as commercial adsorbents for the removal of copper and other heavy metals [15].

Zeolites can be used as ion exchangers in various water treatment applications such as soluble heavy metal removal, water softening and ammonium removal. Zeolites have been widely used as an adsorbent in treating heavy metal because of their effectiveness in adsorbing contaminants [15,16]. They are also extensively used for heavy metal removal from wastewater. However, zeolites can only be generated at temperatures above 500 °C [17,18,19]. Therefore, the high cost of its production at the industrial level requires new, cheaper and less energy-consuming material.

Recently, new geopolymer-based material has been explored in relation to the adsorption of heavy metal [20,21,22]. Geopolymer-based adsorbent possesses exceptional pollutant removal properties [23,24,25]. The geopolymer characteristics have made it a feasible and environmentally friendly option, compared to other materials such as biochar, zeolite and activated carbon, because of its more sustainable production process, which absorbs low energies and by-products [26,27,28]. Kaolin has the potential to be used as a raw material or an aluminosilicate source of geopolymer due to its high content of alumina and silica, which contributes to the formation of geopolymer based on sharing atoms of the (SiO_4_)^4−^ and (AlO_4_)^5−^ that may exist in poly-sialate form, poly sialatedisiloxo and other sialate linkages [29]. Kaolin is one of the naturally occurring clay minerals in the earth’s crust, which is formed by rock weathering [30,31].

The other important physicochemical characteristic of an adsorbent is porosity of geopolymer such as surface area and pore volume [32,33]. These characteristics are significant to increase the efficiency in the removal of copper from aqueous solution, in which the increase in the porous structure of geopolymer will increase the surface area and pore volume, leading to increased adsorption of Cu^2+^ [34,35]. In order to increase Cu^2+^ adsorption, foaming agent should be added during the mixing of aluminosilicate sources and alkali activator [34,35,36,37]. The addition of foaming agent also affects the properties of the geopolymer such as its chemical composition, porosity, morphology, phase and functional group [38]. Some attempts have been made to produce geopolymer using different types of foaming agent such as metal powder (aluminium powder), hydrogen peroxide (H_2_O_2_), and sodium hypochlorite [39,40,41]. Amorphization by mechanical alloying also can improve the porosity of the geopolymer adsorbent [42].

Foaming agents react in various ways to the formation of the geopolymer or alkali activated material. One of the techniques used involves dispersing air through the process of fast stirring, and utilising a surfactant to reinforce the foamed content [43,44]. In oxidation state, metal powders (aluminium powders) react in sodium hydroxide solution and release hydrogen in the process [45]. The reaction equations are as follows [46]:

2*Al* + 6*H*_2_*O* → 2*Al(OH)*_3_ + 3*H*_2_ (1)

*Al(OH)*_3_ + *Na*^+^ + *OH*^−^ → *Na*^+^ + *Al(OH)*_4_^−^ (2)

Aluminium powder is a fast-reacting foaming agent [47]. The structure of kaolin-based geopolymer synthesis with the addition of aluminium powder depends on the Na/Al ratio [48]. The Na/Al ratio in geopolymer slurry was varies from 0.4 to 0.8. A ratio under 1.0 is the best expected ratio because not all alumina is used during the reaction and each negative charge has to be countered by one sodium ion [43]. Foaming agent will enhance the amount of voids inside the material with air content. Thus, increasing the amount of foaming agent will result in decreases in density and yield high specific surface areas [41,49].

Most studies on geopolymer-based adsorbent focused on the raw materials such as fly ash, metakaolin and slag [50,51,52,53]. Until recently, there was a lack of research using kaolin as the starting raw material in geopolymer adsorbent synthesis, although mineral composition in kaolin makes it suitable to be used as a source of raw material, and it can be used as low cost adsorbent. It has been reported that geopolymer offers adjustable porosity for heavy metal removal. Porous geopolymer possesses high specific surface area, making it an ideal candidate for adsorption. Therefore, this study aims to investigate the potential of kaolin-based geopolymer as an adsorbent for Cu^2+^ from wastewater at low synthesis temperature. The addition of foaming agent in the geopolymer is aimed at achieving better understanding of the role of aluminium powder in the production of porous geopolymer and the percentage removal of Cu^2+^ ions.

## 2. Materials and Methods

### 2.1. Raw Material

Kaolin is a clay mineral mainly containing a chemical composition of Al_2_Si_2_O_5_(OH)_4_. In this study, kaolin was used as a starting material for geopolymerization. It consists of very fine particles, with particle size ranging from 90 µm to 120 µm, which contain a large amount of SiO_2_ and Al_2_O_3_. Kaolin was supplied by Associated Kaolin Industries Sdn. Bhd, Petaling Jaya, Selangor, Malaysia.

### 2.2. Synthesis of Geopolymer

In the preparation of geopolymer-based adsorbent, the effect of solid-to-liquid (S/L) ratio and effect of foaming agent are studied. The geopolymer-based adsorbent is prepared by the mixing of raw material with alkali activator using mechanical mixer, as shown in Table 1. After that, the sample will be cured in the oven at temperature, as stated in the table, for 24 h, based on previous research [54]. This process will undergo crushing and sieving to become powder adsorbent with less than 150 µm of particle size.

Based on Table 2, the ratios of 0.6, 0.8, 1.0 and 1.2 wt% aluminium powder were used to determine the optimum foaming agent ratio for kaolin-based geopolymer as inferred from previous study [47,55]. Aluminium powder was added into the mixture of raw material and alkali activator.

All the samples of geopolymer based adsorbents that were prepared were tested for characterization such as phase, functional group, morphology and porosity. These tests were used to determine the change in the mineral peaks, bond arrangement, microstructural development and surface area of geopolymers in assessing geopolymer reaction when added with aluminium powder. The performance of these geopolymer based adsorbents depends on the percentage removal of Cu^2+^, tested by using Atomic Absorption Spectrometry (Perkin Elmer, Llantrisant, UK).

### 2.3. Testing

The concentration of copper was examined using Atomic Absorption Spectrometry (AAS). The chemical compositions of raw materials and geopolymer were determined by using an X-ray fluorescence (XRF) spectrometer (PANanalytical PW4030, MiniPAL 4, Malvern Panalytical, Worcestershire, UK). The MiniPAL-4 model spectrometer uses an energy dispersive detector-controlled instrument. The sample was loaded in the chamber of the spectrometer and operated at a maximum current of 1 mA, which was applied to generate X-ray, and a maximum voltage of 30 kV to stimulate the sample for 10 min pre-set time.

In order to identify the functional group of kaolin and geopolymer-based adsorbent, an FTIR spectrometer (RX1 Perkin Elmer, Llantrisant, UK) was used. Infrared is a useful analytical tool used for both qualitative and quantitative techniques for primarily organic and inorganic materials, while the 1 μm wavelength radiation is used to provide details on vibrational transformations and chemical bonds. Powder samples were analysed using a FTIR-4100, with a scan ranging from 450 to 4000 cm^−1^ and a scan time of 5 min.

Next, phase analysis testing, one of the testing methods used for the characterization of crystalline materials or in the determination of the degree of crystallinity of a compound, was conducted using XRD diffractograms. The phase was characterized for raw material and geopolymer. Samples were collected on an X-Ray Diffractometer (XRD-6000, Shimadzu, Columbia City, IN, US) with CuKα radiation produced at 30 mA and 40 kV at 10–80° at a step size of 0.02°, integrated at a rate of 1.0 s per step.

In addition, morphology analysis was studied by using Scanning Electron Microscope (JSM-6460LA, JEOL, Tokyo, Japan). Information regarding the sample, which includes the surface morphology, can be obtained through the signal derived from the interactions between sample and secondary electron detector. The signals produced during the analysis create a two-dimensional image and disclose details about the sample, including the orientation of the sample materials and the existing morphology (texture).

The surface area and pore structure of geopolymer adsorbent were determined by Brunauer–Emmet–Teller (BET) analysis (TriStar 3000, Micromeritics Instrument Corporation, Norcross, GA, USA). The samples were dried with nitrogen purging or in a vacuum, at elevated temperature. The amount of adsorbed gas is correlated to the total surface area of the particles including pores in the surface.

## 3. Results and Discussion

### 3.1. Characterization of Raw Material

Chemical compositions of the samples were verified through XRF analysis. Table 3 shows the chemical composition of raw kaolin. The SiO_2_ compound was found to exist in kaolin with the highest percentage amounting to 56.4%, followed by Al_2_O_3_ at 37.6% and Fe_2_O_3_ at 2.06%. Other compounds found in kaolin are K_2_O (2.56%), TiO_2_ (0.76%), RuO_2_ (0.17%) and LOI. From the results obtained, kaolin fulfilled the requirement as a precursor of raw material for the manufacturing of the geopolymer. This is due to the fact that materials in forming of geopolymer should be rich in Si and Al as important sources of Si^4+^ and Al^3+^ in binding system that will be activated by alkali activator solution [56,57,58].

**Figure 1:**
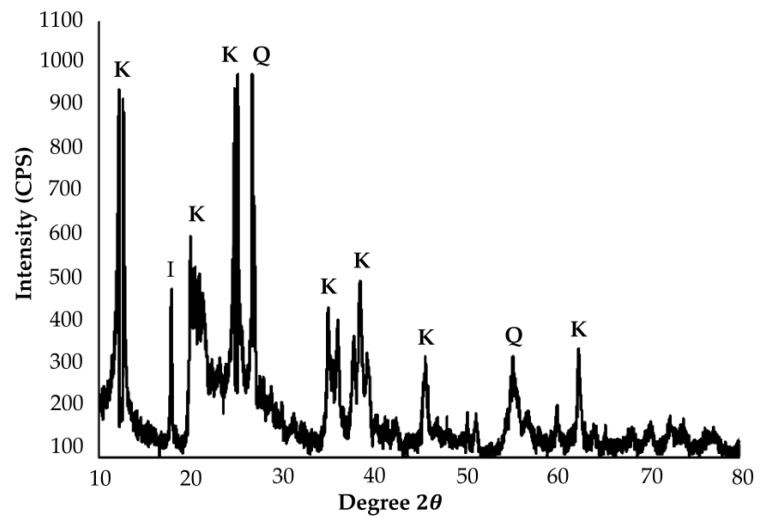
XRD phase of raw kaolin.

The XRD patterns in Figure 1 present the phase analysis for raw kaolin material. From Figure 1, kaolin performed the majority of crystalline phase in the pattern, consisting of kaolinite, quartz and illite. The same phase in the kaolin was found by Worasith [59]. XRD pattern showed that kaolin consists mainly of kaolinite (K) as major minerals together with other minerals such as quartz(Q) and illite (I). The intense peaks associated with kaolinite were at 2θ of 12.1°, 25.2° and quartz at 26.7°. Less intense peaks for kaolinite were observed at 2θ of 19.9°, 35°, 38.5°, 45.6° and 62.4°. Illite, which is a common impurity in kaolin-group minerals, showed existence at 2θ of 17.9°. A less intense peak for quartz was found at 55.1°. This finding is similar to Naghsh and Shams, who stated that the presence of peaks at 13°, 20° and 25° in the raw kaolin show that the major crystalline phase is kaolinite [60].

**Figure 2:**
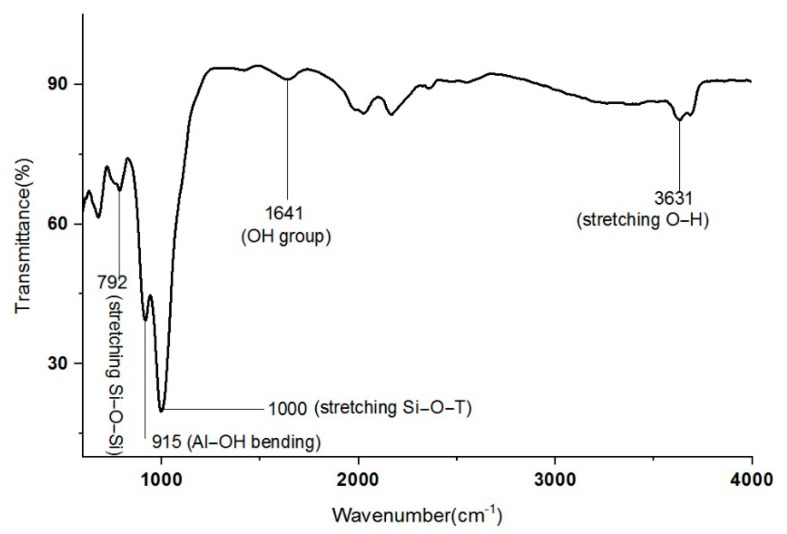
IR spectra of raw kaolin.

Characterization of kaolin using Fourier Transform Infrared Spectroscopy had been conducted to determine the functional group involved in these materials. The IR spectrum of kaolin can be seen in Figure 2. IR spectra were observed at 786 cm^−1^, 916 cm^−1^, 1008 cm^−1^, 3622 cm^−1^ and 3682 cm^−1^. Wavenumber 786 cm^−1^ is attributed to the stretching bond of Si-O-Si, representing quartz from the kaolin. It can be seen that the band at 916 cm^−1^ corresponded to the bending of the Al-OH bond. A peak at the band at 1008 cm^−1^ corresponded to stretching of Si-O or Al-O. The bands at 3682 cm^−1^ and 3622 cm^−1^ were assigned to the O-H vibration of water molecules due to the presence of water in the kaolin. In can be concluded that hydration was performed in the washing and dealkalization stages.

**Figure 3:**
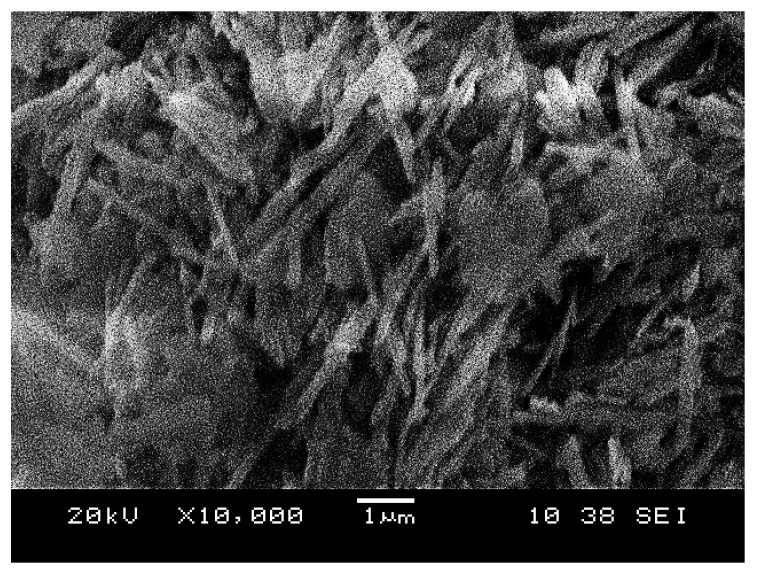
Microstructure of raw kaolin.

Figure 3 illustrates the micrograph of raw kaolin used in this study. It can be seen that kaolin showed particles with morphologies that were flaky and needle like shaped, which were arranged and stacked together. In general, kaolin has an aggregated, edge-to-face and edge-to-edge flocculated composition. The kaolinite crystals that make up most of kaolin deposits are pseudo-hexagonal along with plates, and consist of irregular shaped edges. Saeed found that the micrograph of kaolin clay shows neatly arranged book-like kaolinite particles which are the predominant features of natural soil [61]. However, Worasith found that kaolin contains both platy and tubular shapes which show the presence of kaolinite and halloysite [59]. Zhang stated that different morphologies of kaolin were found at different sources [30].

### 3.2. Effect of Solid-to-Liquid on Adsorption of Cu^2+^

**Figure 4:**
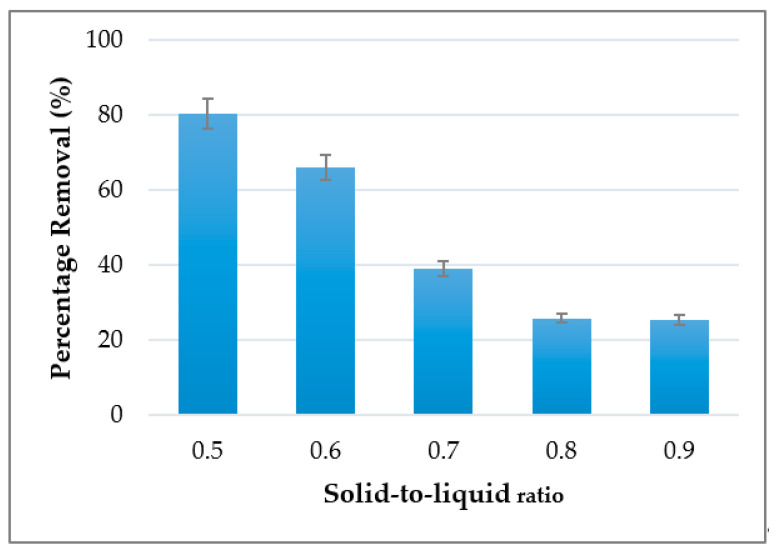
Percentage removal of Cu^2+^ based on effect of solid-to-liquid ratio.

Adsorption of Cu^2+^ ions at different S/L ratios are illustrated in Figure 4. The result demonstrated the potential of kaolin geopolymer in the removal of copper, based on various solid-to-liquid ratios (S/L) of 0.5, 0.6, 0.7, 0.8 and 0.9. Note that the solid-to-liquid ratio of geopolymer at S/L ratio 0.4 was unable to be recorded due to low viscous and limited workability. The trends of the results illustrated in Figure 4 show that the removal of Cu^2+^ decreased as the S/L ratio increased from 0.5 to 0.8. A further increase in S/L will decrease the Cu^2+^ uptake. It was found that the highest percentage removal (80.5%) was achieved at a ratio of 0.5. The graph showed that the removal gradually decreased for the ratios of 0.6, 0.7, 0.8 by about 66.1%, 39.2% and 25.5%, respectively, after which it remained unchanged. This is due to the limitation of binding sites of surface geopolymer adsorbent to bind with the Cu^2+^ ion.

It can be seen that the best S/L ratio for kaolin-based adsorbent to remove Cu^2+^ ion is 0.5. At lower S/L, accelerated dissolution of aluminosilicates is promoted, thus approaching homogeneous mixing and increased geopolymerization reaction. At S/L 0.5, the binding sites of the adsorbent were highest and could be indicative of an ion-exchange mechanism for the removal of Cu^2+^. This finding is in line with those reported by previous researchers [62,63].

**Figure 5:**
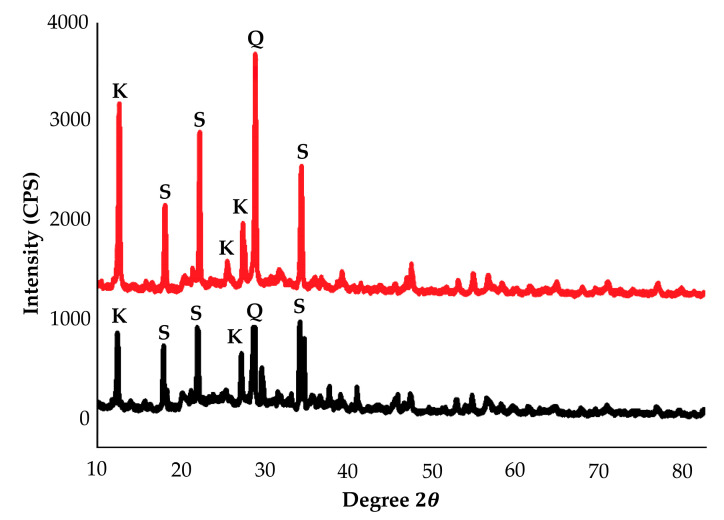
XRD diffractogram of kaolin-based geopolymer at S/L 0.5 and S/L 0.8.

Figure 5 shows the phase analysis of kaolin-based geopolymer adsorbent. The geopolymer was chosen based on the highest and lowest Cu^2+^ percentage removal in the adsorption for the characterization phase. After geopolymerization, the disappearance of the peaks corresponding to kaolinite was observed. This is attributed to the dehydroxylation of the water molecules found in the kaolinite structure by heat treatment. After the activation process, the crystalline phases were dissolved in the alkaline solution and the aluminosilicate phases were developed in the kaolin surface by geopolymerization reaction [64]. The existence of a new phase in the kaolin-based geopolymer indicates the reaction from raw kaolin material to the formation of geopolymer. This demonstrates the creation of a new product with a structure different from that of kaolin. Kaolin-based geopolymer at a ratio of 0.5 appeared to be more intense in peaks compared to that at a ratio of 1.0. The major peaks associated with kaolinite were found at 12.6°, 25°, 26.7° and 46.2°. Quartz showed an appearance at 28.1°. Sodalite peaks appeared at 17.8°, 21.7° and 33.4°. Sodalite peaks indicate the geopolymerization reaction between Na, SiO_2_ and Al_2_O_3_.

**Figure 6:**
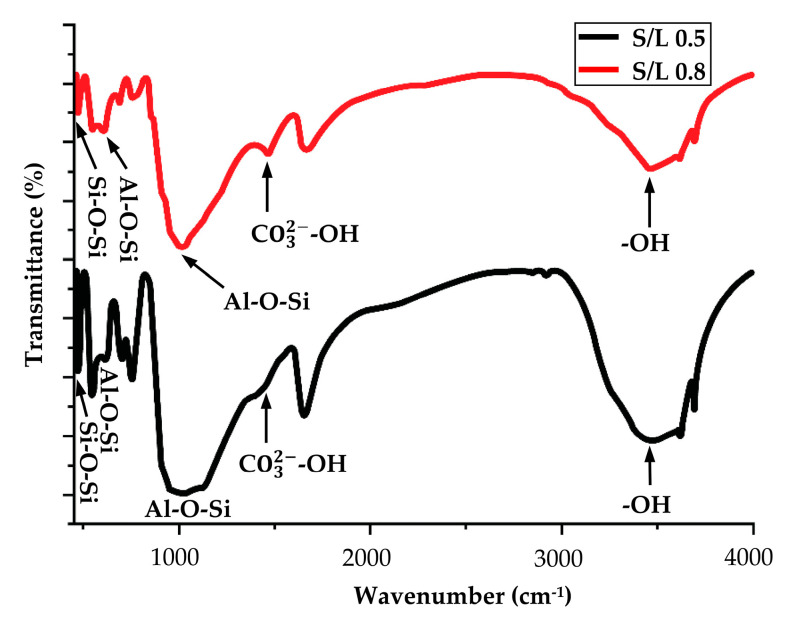
IR spectra of kaolin-based geopolymer at S/L 0.5 and S/L 0.8.

Figure 6 shows the FTIR transmittance spectra of kaolin geopolymer for solid-to-liquid (S/L) ratios of 0.5 and 0.8. Geopolymerization caused the change in the chemical environment and chain structure of Si-O bond in kaolin (1008 cm^−1^, from Figure 1), moving to the formation of Al-O-Si bonds (1000 cm^−1^) of the geopolymer after curing temperature was reached. This shows that the solidification process that occurs is a chemical reaction of the alkali activator solution reacting with aluminosilicate raw material, which produces generation of a new substance. The bands at 600 cm^−1^ were due to Al-O-Si stretching vibration and the presence of the peak at 450 cm^−1^ is due to the Si-O-Si bending vibration. For the geopolymers, the characteristic peaks at approximately 3450 cm^−1^ and 1650 cm^−1^ were attributed to stretching and bending vibrations of hydroxyl due to the presence of water in the geopolymers. Spectrums showed a different peak at wavenumber 1450 cm^−1^, referring to CO_3_^2−^ ions. Samples with high R/Al (R: Na or K) ratios tend to exhibit more pronounced carbonate ions vibrations. Therefore, broad peak was shown for the sample S/L ratio of 0.5 compared to the S/L ratio of 0.8, which means that sample S/L ratio of 0.5 produces more CO_3_^2−^ ions due to the higher content of Na^+^ in the sample. The shifting and reduction of peaks in FTIR spectrum confirms the formation of a poly(sialate-siloxo) chain in the structure by geopolymerization reaction.

**Figure 7:**
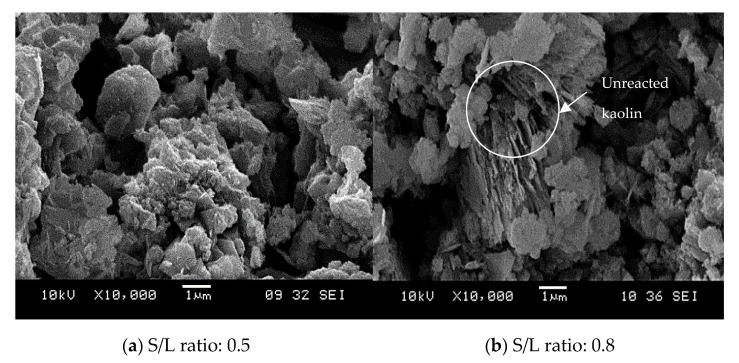
Microstructure of kaolin-based geopolymer: (**a**) S/L ratio: 0.5, (**b**) S/L ratio: 0.8.

Figure 7 shows the microstructure of geopolymer with different solid-to-liquid ratios (S/L). The geopolymer adsorbents with lowest and highest adsorptions of Cu^2+^ from wastewater were selected. After the geopolymerization reaction, the morphology of the geopolymer is a network of polysilicate layers produced by the disappearance of kaolinite particles. SEM analysis showed that the particles were amorphous agglomerates, indicating that raw materials are dissolved under alkaline conditions and a new amorphous structure was formed during geopolymerization [65,66,67]. Geopolymer matrix in Figure 7a showed a loose grained structure and coexistence of the geopolymer gel. Figure 7b shows that the microstructure contains unreacted raw material. These are due to the unreactive particles of kaolinite in the raw material which remained in the geopolymer paste. An increase in the S/L ratio will decrease the alkali activator added during the mixing of geopolymer. Therefore, kaolinite particles of kaolin were undissolved in the geopolymerization reaction.

Physical characterization of the surface area of adsorbent was executed through BET test by using N_2_ as adsorbate. Table 4 shows the result of the surface area analysis for geopolymer adsorbents. Results showed that an increase in the aluminosilicate raw material will decrease the porous surface area of the geopolymer. The pore volume of geopolymer adsorbent remained unchanged in both samples. The surface area decreased from 23.58 m^2^/g to 20.32 m^2^/g. This means that the S/L ratio of 0.5 leads to higher adsorption sites compared to the S/L ratio of 0.8. Larger surface area may have facilitated the diffusion of Cu^2+^ into the internal network of the adsorbent.

### 3.3. Effect of Foaming Agent on Adsorption of Cu^2+^

**Figure 8:**
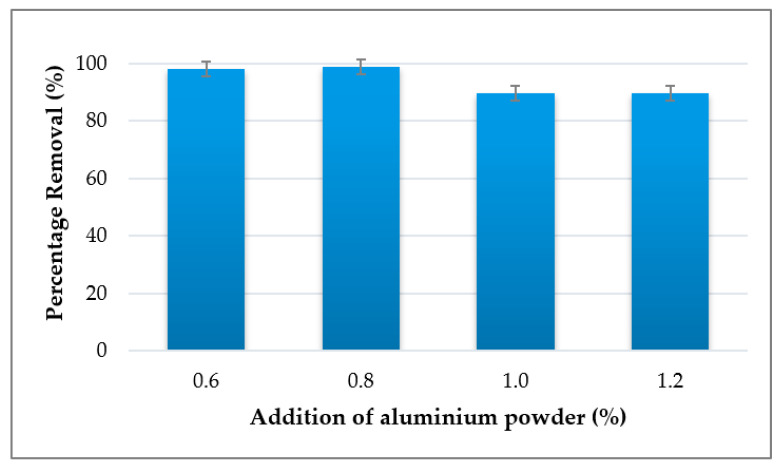
Percentage removal of Cu^2+^ at different percentages of aluminium powder addition.

In order to improve the porosity of geopolymer and copper absorption by geopolymer adsorbent, aluminium powder was used as a foaming agent. As presented in Figure 8, the effect of adding a foaming agent to the kaolin based geopolymer was analysed in terms of heavy metal uptake by kaolin-based geopolymer adsorbent. Aluminium powder was used as foaming agent in the kaolin based adsorbent to enhance the porosity in the adsorbent by generating hydrogen gas. The highest removal was found at 0.8% addition of aluminium powder with 98.7% removal. The increment in the Cu^2+^ adsorption at this ratio indicates the improvement in the efficiency of the geopolymer adsorbent. Good percentage removal was also obtained at 0.6% Al powder. However, the utilization did not achieve the highest percentage removal. The removal efficiency of the adsorbent declined with the increase in aluminium powder doses of 1.0% and 1.2% where the amount of removal was 89.7% for both. This may be due to excessive aluminium being bonded with silica. Furthermore, this indicates that the coordination number of aluminium in the materials will eventually have an effect on its bonding in the matrix [67].

**Figure 9:**
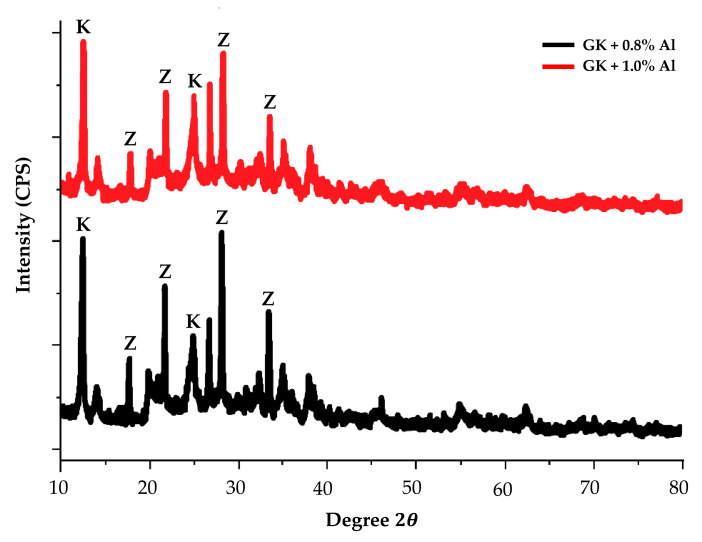
XRD diffractogram of kaolin geopolymers with different percentages of aluminium powder addition.

Figure 9 shows the IR spectra for the effect of foaming agent on the kaolin-based geopolymer. The addition of foaming agent (Al powder), will reduce the amount of alkali activator solution in the geopolymer, thus enhancing the more crystalline phase structure in the geopolymer adsorbent. The porous structure of geopolymer contains high alumina and silica. Based on Figure 9, an increase in the percentage of aluminium powder in the kaolin-based geopolymer does not produce significantly different peaks in the formation in the XRD diffractogram. This indicates that the weight percentage difference of Al powder added to the geopolymer does not affect the geopolymerization reaction. XRD diffractogram showed that formation of zeolite phase was obtained at peaks 17.5°, 22.1°, 28.6° and 34.5°.

**Figure 10:**
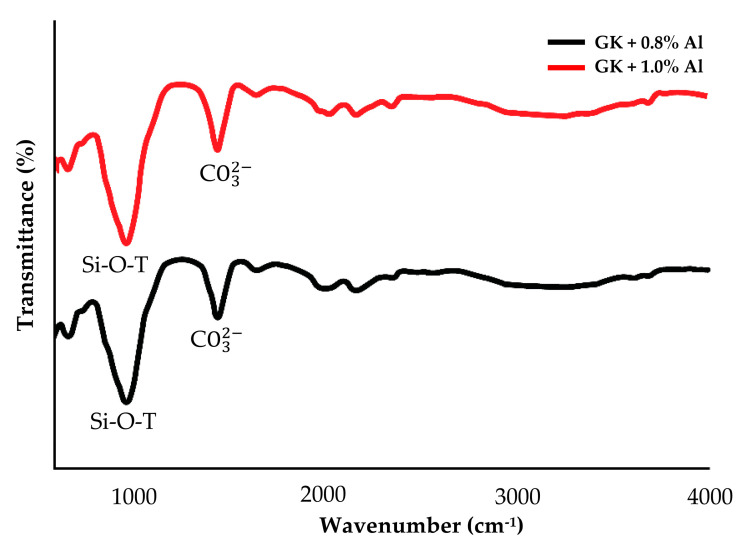
IR spectra of kaolin geopolymers with different percentages of aluminium powder addition.

FTIR results of kaolin-based geopolymer with the addition of aluminium powder are presented in Figure 10. The absence of a peak at 3695 cm^−1^ indicates completion of the process of dehydroxylation of the OH group [68]. The peak at 973 cm^−1^ in the plot corresponds to the stretching of Si-O-T where T can be Si or Al. Carbonate ions (CO_3_^2−^) appeared at the wavenumber peak at 1445 cm^−1^, as exhibited by Na content in the sample. In hydroxide activated geopolymers, there is preferential dissolution of Al from kaolin at the beginning of the reaction. Therefore, the concentration of Si in the solution is initially less than that of Al, which is believed to create the induction time identified in these systems. The appearance of Al powder in solution is because Al reaction in the sample could minimize the driving force for further dissolution of Al particles and shift the sequence of dissolution of Al and Si species from kaolin. The positioning of the Si-O-T band reveals some details on the extent of the contribution of Al and Si to the formation of gel. The gel with the higher Si amount indicates bands with higher wavenumbers, and, with more Al participations, the band moves to lower wavenumbers. Despite the huge availability of Al in the study, the position of the main band is identical for both samples after hours of reaction.

**Figure 11:**
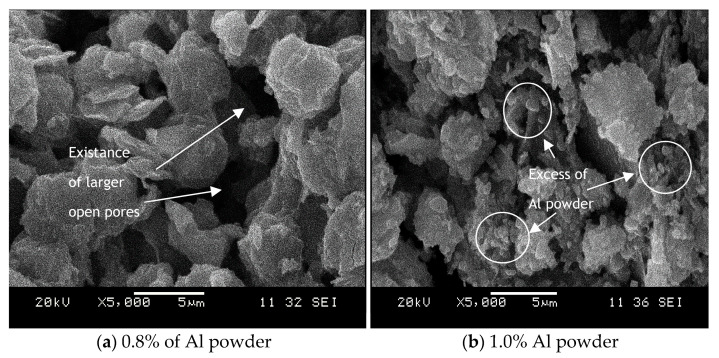
Effect of foaming agent on microstructure of kaolin-based geopolymer: (**a**) 0.8% Al powder, (**b**) 1.0% Al powder.

Figure 11 illustrates the microstructure of geopolymer adsorbent after being added with aluminium powder at 5000× magnifications. Figure 11a represents kaolin-based geopolymer adsorbent with addition of 0.8% aluminium powder, while Figure 11b, represents kaolin-based geopolymer adsorbent with the addition of 1.0% aluminium powder. An addition of 0.8% Al powder generates larger pores that are homogeneous in size and distribution. As seen in Figure 11, the adsorbent in Figure 11a looks more homogeneous compared to the one in Figure 11b. In Figure 11b, some unreacted materials appeared in the matrix which may be due to the injection of aluminium powder which has achieved its optimum. These results confirmed that the different amounts of Aluminium powder added to the geopolymer paste can influence the microstructure of geopolymer adsorbent. From the results obtained and the highest adsorption of heavy metals, the optimum ratio additions of Aluminium powder at 0.8 percent showed better porosity of geopolymer adsorbent. The release of aluminium into the solution as a result of the metallic reaction contributes to better interaction of unreacted particles and better microstructural growth [46].

The results of BET analysis for kaolin-based geopolymer adsorbent with the addition of foaming agent are summarized in Table 5. From Table 5, the results showed that the addition of foaming agent increased the porosity surface area of geopolymer adsorbent. The addition of Al powder in kaolin-based geopolymer yields a specimen with lower bulk density and higher apparent porosity [48,55]. The surface area obtained was 54.81 m^2^/g and the pore volume was 0.049 cm^3^/g for the addition of Al powder at 0.8%. At 1.0% Al powder, the surface area of kaolin-based geopolymer increased to 52.08 m^2^/g and the pore volume decreased to 0.032 cm^3^/g. Therefore, the addition of the foaming agent will increase the surface area and pore volume. In addition, the pore volume distribution of geopolymers was observed to improve to larger pores as the Si/Al ratio increased, indicating that the soluble silicon content would lower the number of geopolymers. Adsorption performance and adsorption rate increased as the porosity of geopolymer adsorbents increased [69].

### 3.4. Adsorption Study of Cu^2+^

**Figure 12:**
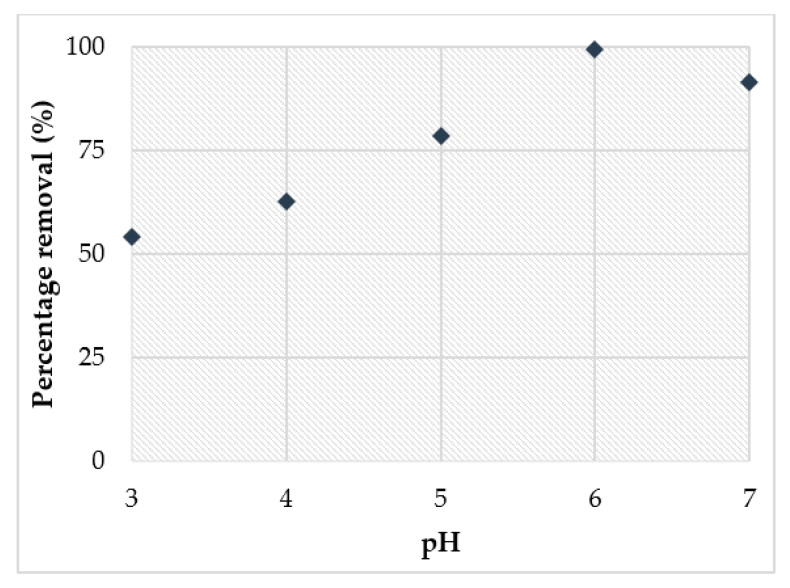
Effect of pH on percentage removal of Cu^2+^.

The influence of pH is an important factor for the removal of heavy metal from water. The initial pH 3-7 were used to study the effect of pH as the solution in removing copper ions (Cu^2+^) through aluminated kaolin-based geopolymer. Figure 12 illustrates the effectiveness of Cu^2+^ with the initial pH 3-7 in the adsorption process. Results showed that the metal removal was dependent on pH solution. The concentration of Cu^2+^ in the solution decreased as the initial pH of the solution increased which indicates the increment of the removal efficiency of Cu^2+^ being adsorbed into geopolymer. The amount of Cu^2+^ removed during the adsorption process increased from 56% to 99.1% when pH was increased from 3 to 6. The removal of Cu^2+^ was affected by the change in the pH of the solution. In the acidic medium, the surface of the geopolymer was surrounded by H^+^ ions which limit the interaction of the solute ions (Cu^2+^) with the surface sites. On the contrary, in the basic medium, the concentration of H^+^ ions decreased, inducing a strong contact with the Cu^2+^ ions and the surface sites. Some studies had reported findings related to Cu^2+^ adsorption habits. At lower pH, the adsorption sites were saturated with H^+^ and the adsorption of Cu^2+^ ions was low; as the pH increased, the adsorption sites became accessible and the adsorption of copper ions increased [70].

At low alkalinity, more positive species of H_3_O^+^ ions are accessible in a solution that competes with the positive Cu^2+^ ions present as active sites on the geopolymer surface. With an increase in pH, less H_3_O^+^ is available, contributing to improving the connectivity of Cu^2+^ to active sites. This was the predicted pattern for the impact of pH on metal adsorption, where adsorption increases to a certain value with the increase in pH values, and then decreases with a further increase in pH. It is known that increasing pH of the solution to value higher than 6 would favour the precipitation of Cu^2+^ as Cu(OH)_2_ [38,70,71]. The highest removal of copper was obtained at pH 6 with the removal of about 99%.

**Figure 13:**
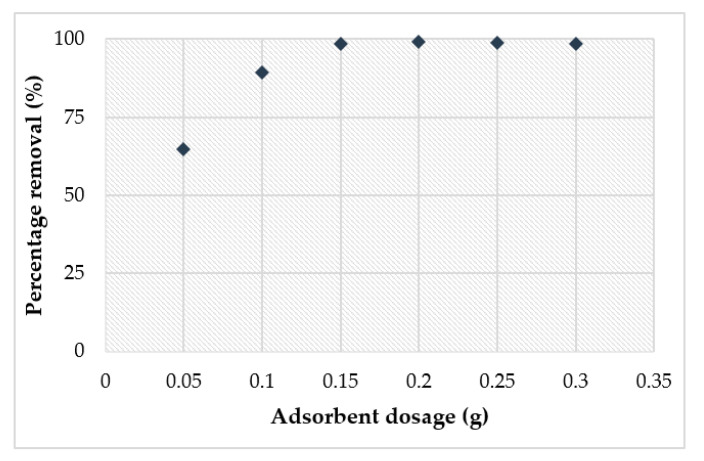
Effect of adsorbent dosage on percentage removal of Cu^2+^.

Figure 13 presents the results indicating that the removal efficiency increased as adsorbent dosage increased from 0.05 g to 0.3 g. This indicates that at low adsorbent dosage, the available active sites are insufficient to take up all available Cu^2+^ ions in the solution. A sharp increase in removal efficiency was noticed as the synthesized geopolymer dose increased from 0.05 to 0.1 g, while an insignificant increase was noticed as the dose increased from 0.15 to 0.3 g. Adsorbent mass optimization is a very critical parameter for adsorbent power management. This points to the fact that a dosage of 0.15 g is the optimal adsorbent value for the fastest removal of Cu^2+^. If the geopolymer dosage increases, the vacant site accessible for binding Cu^2+^ ions increases, resulting in greater adsorption efficiency.

**Figure 14:**
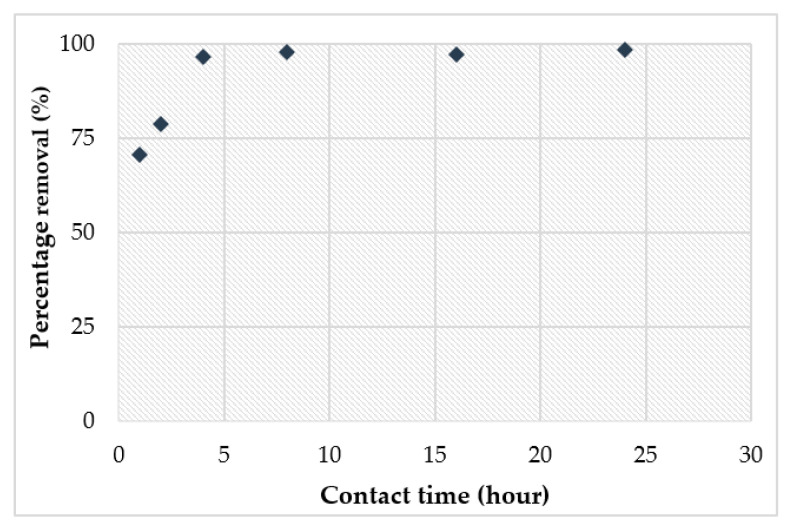
Effect of contact time on percentage removal of Cu^2+^.

The data pertinent to the effect of varying contact times on the Cu^2+^ copper efficiency are shown in Figure 14. The time dependent behaviour of Cu^2+^ adsorption was tested by adjusting the contact time from 2 to 24 h. The results demonstrated that the percentage removal of Cu^2+^ was rapid during the first 4 h. Afterwards, the equilibrium time was reached within 5 h for 99% removal, respectively. No significant difference in the removal percentage of the geopolymer was observed after equilibrium. The time consumed for adsorption of Cu^2+^ metal ions depended on the geopolymer achieving saturation within a specific time period. After 4 h of contact time, a major removal of Cu^2+^ took place. The highest removal of Cu^2+^ ions was 99.6%. Removal of Cu^2+^ from the solution reached an equilibrium within 4 h.

## 4. Conclusions

The effect of aluminium powder on kaolin-based geopolymer adsorbent for removal of Cu^2+^ has been investigated. Based on the results obtained after addition with aluminium powder, the following can be concluded:

- The XRD diffractogram indicates the presence of zeolite peaks of kaolin-based geopolymer which were obtained at 80 °C, which is lower than the sintering temperature for conventional zeolite.
- IR spectra indicate that kaolin-based geopolymer dehydroxyled the OH group completely, consequently increasing the active surface area to adsorb Cu^2+^.
- The porous structure in the geopolymer adsorbent is attributed by increase in surface area from 23.58 m^2^/g to 54.81 m^2^/g.
- The morphology showed that the geopolymer adsorbent contains a well-developed porous surface area. Metallic reaction from aluminium powder contributes to better interaction of unreacted particles and increased microstructural growth. Thus, increased geopolymerization reaction will homogeneously produce geopolymer paste, consequently increasing the rate of copper adsorption.
- The adsorption study showed that the highest removal of Cu^2+^ (98%) obtained at pH 6 achieved the optimum adsorbent dosage at 0.15 g within 4 h.

Further improvement can be done by studying the desorption of the Cu^2+^ ion from kaolin-based geopolymer adsorbent with a focus on the regeneration and reuse of the adsorbent. In addition, an adsorption study for different types of heavy metal ions such as Pb^2+^, Cd^2+^, As^3+^, Hg^2+^ and etc. could be conducted in the future. This work can further be used as a suspension and solidification method of synthesis and to investigate mechanical properties such as compressive strength according to standard ASTM C109.

## Figures and Tables

**Table 1:** Mix-design of solid-to-liquid used in this study.

Parameter	Kaolin
Solid-to-liquid ratio	0.5, 0.6, 0.7, 0.8, 0.9
NaOH-to-Na_2_SiO_3_ ratio	1.0
Curing Temperature	80
Molarity of NaOH	10
Sieve size	<150 µm

**Table 2:** Mix-design of foaming agent ratio for kaolin-based geopolymer.

Parameter	Kaolin-Based Geopolymer
Foaming agent (wt%)	0.6, 0.8, 1.0, 1.2
Alkali activator	S/L ratio 0.5, Na_2_SiO_3_/NaOH ratio 1.5
Sieve size	<150 µm

**Table 3:** Chemical composition of raw kaolin.

Element	Kaolin (%)
SiO_2_	56.4
Al_2_O_3_	33.6
Fe_2_O_3_	4.06
K_2_O	3.48
TiO_2_	0.76
RuO_2_	0.17
ZrO_2_	0.08
LOI	1.45

**Table 4:** Surface area and pore volume of kaolin-based geopolymer at different S/L ratios.

Properties	Kaolin Geopolymer	
S/L ratio	0.5	1.0
Surface area (m^2^/g)	23.58	20.32
Pore volume (cm^3^/g)	0.05	0.051

**Table 5:** Surface area and pore volume of kaolin-based geopolymer with different percentages of aluminium powder addition.

Properties	GK + 0.8% Al	GK + 1.0% Al
Surface area (m^2^/g)	54.81	52.08
Pore Volume (cm^3^/g)	0.049	00.32

## Data Availability

The data presented in this study are available on request from the corresponding author.
